# The STRESS-EU database: A European resource of human acute stress studies for the worldwide research community

**DOI:** 10.1016/j.nsa.2024.104063

**Published:** 2024-04-04

**Authors:** Milou S.C. Sep, Kim Veenman, Christiaan Vinkers, STRESS-EU consortium, Consortium members, Milou S.C. Sep, Kim Veenman, Philippe C. Habets, Valeria Bonapersona, Patricia Bakvis, Ulrike Bentele, Elisabeth Binder, Susan J.T. Branje, Tanja Brückl, Sandra Cornelisse, Philip Dickinson, Bernet M. Elzinga, Andrea W.M. Evers, Guillén Fernández, Elbert Geuze, Catharina A. Hartman, Erno J. Hermans, Dennis Hernaus, Marian Joëls, Reinoud Kaldewaij, Wim H.J. Meeus, Maria Meier, Henriët van Middendorp, Stefanie A. Nelemans, Nicole Oei, Tineke Oldehinkel, Jacobien van Peer, Jens Pruessner, Conny Quaedflieg, Karin Roelofs, Susanne R. de Rooij, Lars Schwabe, Tom Smeets, Victor Spoormaker, Marieke S. Tollenaar, Rayyan Tutunji, Anna Tyborowska, Christiaan Vinkers

**Affiliations:** a Department of Psychiatry, Amsterdam University Medical Centers Location Vrije Universiteit Amsterdam, the Netherlands; b GGZ inGeest Mental Health Care, Amsterdam, the Netherlands; c Amsterdam Neuroscience, Mood, Anxiety, Psychosis, Sleep & Stress Program, Amsterdam, the Netherlands; d Amsterdam Public Health, Mental Health Program, Amsterdam, the Netherlands; e Leiden University, the Netherlands; f Utrecht University, the Netherlands; g University of Konstanz, Germany; h Max Planck Institute of Psychiatry, Germany; i University of Amsterdam, the Netherlands; j McGill University, Canada; k Radboud University Medical Center, the Netherlands; l University Medical Center Utrecht, the Netherlands; m University Medical Center Groningen, the Netherlands; n Maastricht University, the Netherlands; o Linköping University, Sweden; p Radboud University, the Netherlands; q Amsterdam University Medical Center, the Netherlands; r Tilburg University, the Netherlands; s Department of Anatomy & Neurosciences, Amsterdam University Medical Centers, Vrije Universiteit Amsterdam, the Netherlands

## Abstract

Our current understanding of the human stress response and its role in health, resilience, and (psycho)pathology stems largely from acute stress studies in controlled laboratory settings. Comparability of findings across these individual studies is comprised, as sample size are often small, between-individual variation in the stress response is large and variation in stress-induction procedures and measurement timing is substantial. To overcome this, 16 research groups across Europe have established the STRESS-EU database. A unique resource with individual participant data (*n* = 6576) of acute stress studies to promote data reuse and facilitate both meta-analytical and proof-of-principle analyses with high statistical power, that can be hypothesis- or data-driven. This short communication highlights the structure, content, access and contribution procedures and future plans of the STRESS-EU database and invited researchers worldwide to contribute to this data resource.

## 1 Background – *Stress research to understand health, resilience and (psycho)pathology*

Actual or anticipated physical and psychological challenges (“stressors”) trigger a complex cascade of (neuro)biological, emotional, cognitive and behavioral changes known as the ‘stress response’ (de Kloet et al., 2005; Joëls et al., 2009, 2012; Hermans et al., 2014; van Oort et al., 2017; Russell et al., 2019). There is a central role for the hypothalamus-pituitary-adrenal (HPA) axis and its end product cortisol within this response, which is modulated by both individual (e.g. genetics, sex, age, personality, previous life experiences, etc.) and stressor (e.g. duration, intensity, controllability) related factors (Mcewen et al., 2020; Sanacora et al., 2022; Oyola et al., 2017; Juruena et al., 2020; Sze et al., 2020; Mikneviciute et al., 2023; Kuhn et al., 2021; Xin et al., 2020; Kühnel et al., 2020).

A healthy stress response is dynamic and prepares an individual to quickly and adaptively respond to a stressor and recover thereafter (de Kloet et al., 2005; Joëls et al., 2009; Russell et al., 2019; Mcewen et al., 2020; Richter-Levin et al., 2021). However, both exposure and responses to stressors are also related to (psycho)pathology (Agorastos et al., 2022; Cohen et al., 2016; Rantala et al., 2021; Soravia et al., 2006). Severe, repeated or chronic stressors can affect brain health (Russell et al., 2019; Mcewen et al., 2020; Lupien et al., 2009, 2018; Wekenborg et al., 2019), and alterations in the (cortisol) stress response are related to physical and mental health (Turner et al., 2020); for example in patients with cardiovascular diseases (Kuckuck et al., 2024), multiple sclerosis (Kern et al., 2014), current mood or anxiety disorders (Zorn et al., 2017) and other psychiatric disorders (van Oort et al., 2020). Importantly, the relations between stress and psychopathology or resilience differ considerably between individuals (e.g. see (Zorn et al., 2017; Schäfer et al., 2022)). There is a long scientific tradition of research into the (human) stress response and understanding its role in health, resilience, and (psycho)pathology (Richter-Levin et al., 2021; Godoy et al., 2018; Kalisch et al., 2017; Bhatnagar, 2021).

Our current understanding of the human stress response largely stems from studies in controlled environments, typically laboratories, where acute stress is experimentally induced using (different versions of) well known emotional, physical and social-evaluative stress-inducing paradigms (Bonapersona et al., 2022; Dickerson et al., 2004). The aversive viewing paradigm (AVP) (Hermans et al., 2011; Henckens et al., 2009) is an example of an emotional paradigm and the Cold Pressor Test (CPT) (Silverthorn et al., 2013; Hines et al., 1932) an example of physical paradigm. Examples of social evaluation paradigms include the (individual or group-based) Trier Social Stress Test (TSST) (Kirschbaum et al., 1993; von Dawans et al., 2011; Allen et al., 2017), the socially-evaluated CPT (SECPT) (Schwabe et al., 2008, 2018), and the Maastricht Acute Stress Test (Smeets et al., 2012). There are also online (Gunnar et al., 2021; Meier et al., 2022) and virtual reality (VR)-based stress tests (Shiban et al., 2016; Zimmer et al., 2019). These studies typically monitor the (stress) response to an emotional, physical or social/psychological stressor with salivary cortisol measurements as biomarker for HPA-axis activity (Hellhammer et al., 2009).

Limiting the comparability of findings across individual acute stress studies is that they are quite heterogeneous due to small sample sizes, large inter-individual differences in the stress response, and variability in stress-induction procedures and the timing of outcome measurements (Richter-Levin et al., 2021; Bonapersona et al., 2022; Herbison et al., 2016; Foley et al., 2010; Simon et al., 2022). The collaborative and interdisciplinary STRESS-NL database consortium (with 12 research groups from six Dutch universities) was founded to overcome these challenges and progress our understanding of the complex human stress response in health and disease (Bonapersona et al., 2022). Over the last years, a collaboration of 16 research groups across Europe (from nine universities) led to the expansion of this initiative into the STRESS-EU database. Here, we aim to highlight the benefits of this expanded database for the international community of stress researchers.

## 2 Objectives and benefits of the STRESS-EU database

The objective of the STRESS-EU database (www.stressdatabase.eu) is to accelerate our understanding of the human acute stress response in health and disease by creating a unique framework to promote data reuse and facilitate both meta-analytical and proof-of-principle analyses with high statistical power that can be hypothesis- or data-driven. The international database combines (neuro)biological, physiological, and behavioral data from laboratory-based human studies that used acute stress tests and builds on the infrastructure of the STRESS-NL database, which has been previously described (Bonapersona et al., 2022). The database is open to all qualified researchers worldwide and supported by the STRESS-NL consortium (www.stress-nl.nl) and the Resilience Network of the European College of Neuropsychopharmacology (ECNP) (www.ecnp.eu/research-innovation/networks-thematic-working-groups/list-ecnp-networks/resilience). Below we will highlight the status of the database and describe the process for data access and contribution.

## 3 Collaborative efforts and database composition

**Fig. 1:**
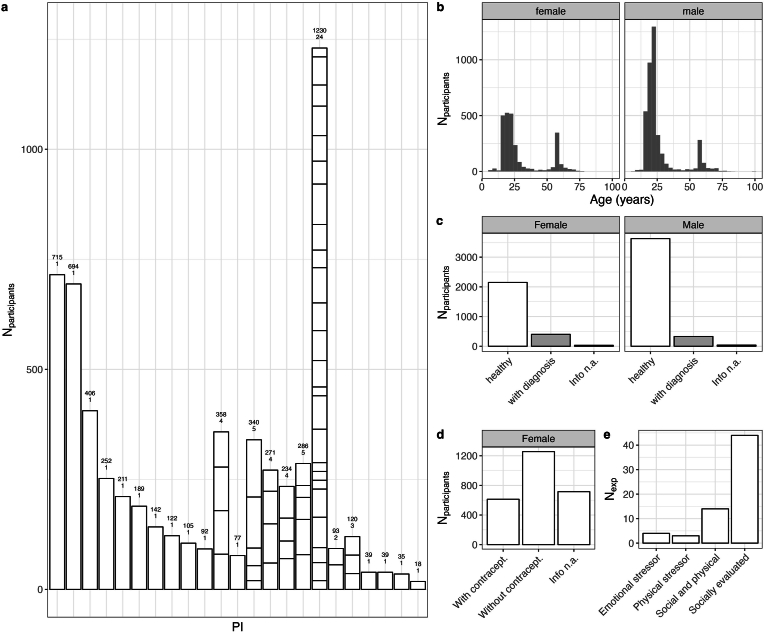
Demographics, population and stress-induction paradigms. *A) Numbers of participants across principal investigators (PI's), height equals the number of participants per study, stacked by PI. Upper number above bar = number of participants; lower number above bar = number of experiments. B) Distribution of age across females and males. C) Count of diagnoses across females and males. D) Information about contraceptive use in females. E) Number of experiments per type of stress-induction paradigm.* This figure is updated with permission from (Bonapersona et al., 2022).

The STRESS-NL database (Bonapersona et al., 2022) was transitioned into the STRESS-EU database and further expanded under this name. In 2021, the former STRESS-NL database included individual participant data (IPD) from 5529 participants from 57 experiments from 12 Dutch research groups at six different universities (Bonapersona et al., 2022). The current STRESS-EU database now includes IPD from 66 acute stress experiments (from 50 datasets) from 16 international research groups from 9 different universities. It includes data of 6576 participants, of which 2584 are female and 3992 are male (Fig. 1A). These participants have an age range between 6 and 99 years (females: mean[*SD*] = 29.1[+-16.49]; males: mean[*SD*] = 26.05[+-13.70]). Age is bimodally distributed, with a clear peak around 20 years (Fig. 1B). This over-representation of young adults is due to a substantial number of studies that recruited students as participants. The STRESS-EU database includes participants described as healthy individuals (87%) and participants with a past or current diagnosis (13%) (neurological, psychiatric or physical, Fig. 1C). Information on contraceptive use (Fig. 1D) and menstrual phase is available for respectively 72% and 22% of the female participants.

## 4 Diversity in research methods and data

*Stress-induction paradigms.* Different types of acute stress tests are available in the STRESS-EU database (Fig. 1E). These behavioral paradigms can be roughly categorized into the following categories: emotional (e.g. AVP), physical (e.g. CPT), socially evaluated (e.g. TSST, Leiden Public Speaking Task (PST) (Westenberg et al., 2009)) and combinations of multiple paradigms (e.g. SECPT, MAST, etc.). Of the participants in the STRESS-EU database 78% were exposed to stress tests and 22% of participants were exposed to a non-stressful control condition (note, some participants were exposed to stress and control tests in a cross-over design).

**Fig. 2:**
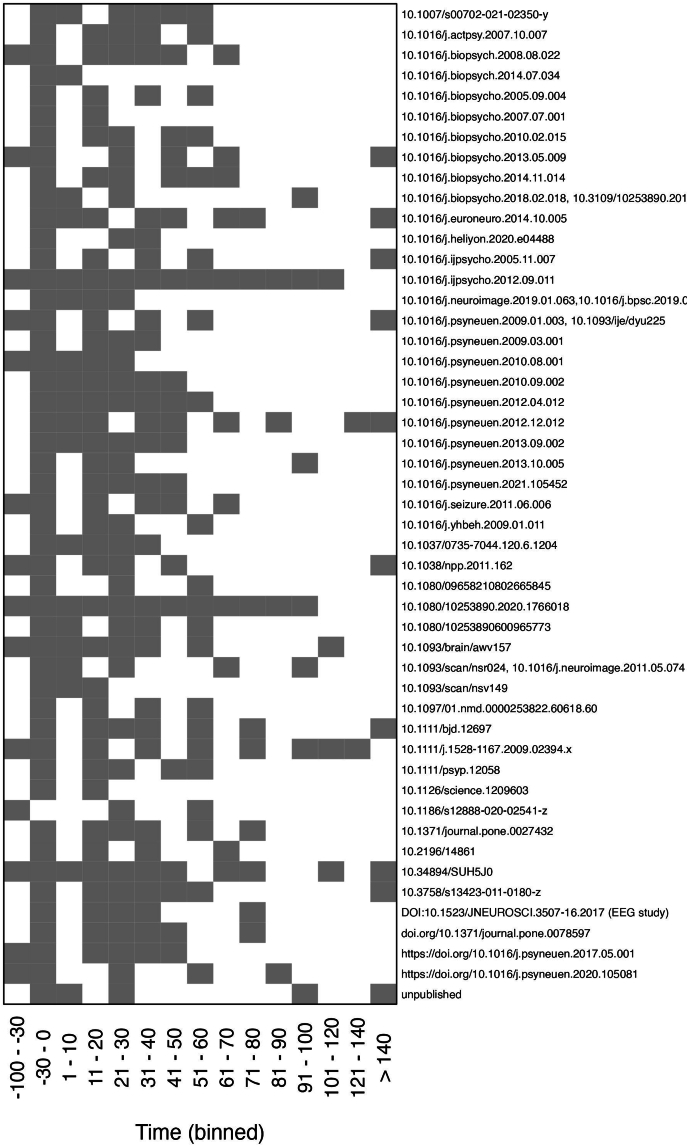
**Heat map of cortisol time points across experiments.** The x-axis depicts time bins with respect to stressor onset (time = 0) and the DOI's on the y-axis correspond to the current studies included in the STRESS-EU database. *Grey = measurement available, white = measurement absent.* This figure is updated with permission from (Bonapersona et al., 2022).

*Stress-related outcomes.* The common outcome measure in all current studies in the STRESS-EU database is salivary cortisol concentration. This is the main (required) variable, to contribute data for the database. Although cortisol is only one of many biomarkers responding to acute stress (Joëls et al., 2009), it is a strong responder together with the autonomic nervous system (Ulrich-Lai et al., 2009) and most commonly measured in acute stress research (Dickerson et al., 2004; Foley et al., 2010); making it a very useful variable to connect information across studies in the STRESS-EU database. Cortisol data was collected between 2 and 20 time points across studies, with a mean of 5.3 (Fig. 2). Most studies collected cortisol data in the afternoon (67%), 25% of studies collected data in the morning, and 9% collected a combination of the two. Baseline cortisol data was measured in almost all experiments. The earliest cortisol collection was 90 minutes before the start of the stress test, and the latest collection was 140 minutes after the start of the stress test. There was one exception of collecting cortisol 24 hours later.

**Fig. 3:**
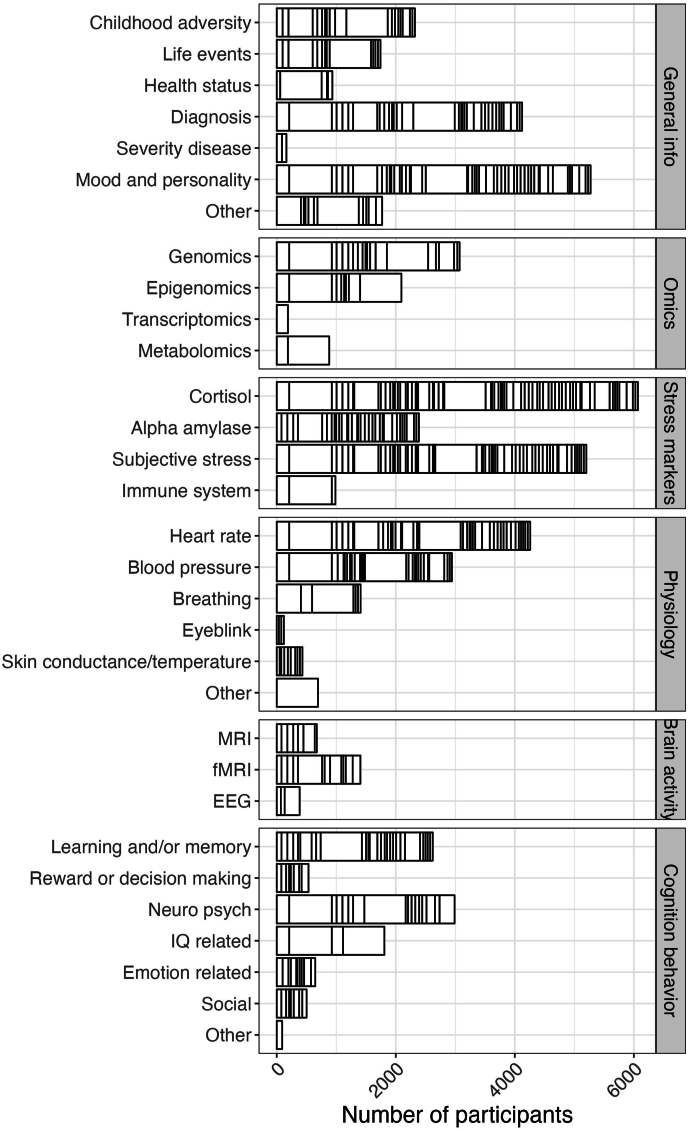
**Participants across outcome measures.** Number of participants (stress and control groups) per (grouped) outcome measure. Each rectangle in the frequency bar plots represent a unique study. Of note, individual participant cortisol values are available for all studies. This figure is updated with permission from (Bonapersona et al., 2022).

The STRESS-EU database centrally stores a limited selection of anonymized IPD, such as demographic variables (age, sex, education, etc.) and primary stress-related outcomes (e.g., cortisol, alpha-amylase and subjective stress ratings). The database further contains meta-data about other secondary stress-related outcomes and correlates that were collected by individual studies (Fig. 3). This data is also available to approved analysis plans (via the study PIs) and includes for example physiology (e.g. blood pressure, heart rate), omics, questionnaires (e.g. childhood trauma, life events, overall health), (epi)genetics, brain structure and function (e.g. MRI and EEG), and cognitive and behavioral tests (e.g. IQ, attention, anxiety). Meta-information about these outcomes can be explored on the online portal of the STRESS-EU database (www.stressdatabase.eu).

## 5 Data access and contribution

**Fig. 4:**
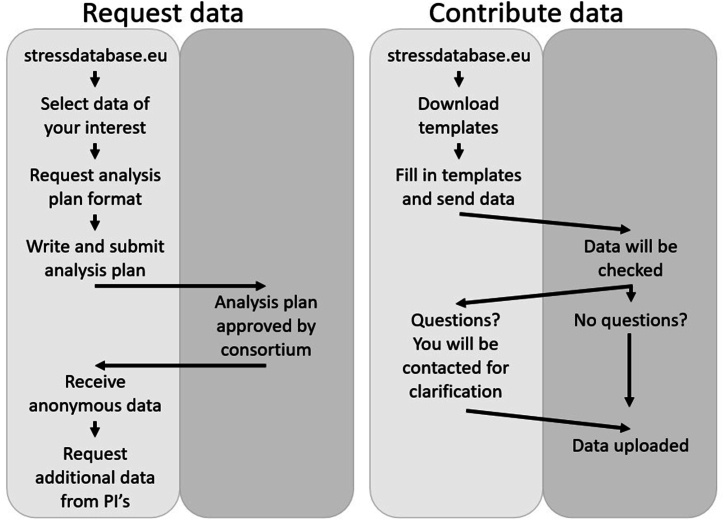
**Flowchart how to request or contribute data.** Light gray: actions by researchers; dark gray: actions by data manager STRESS-EU database.

The STRESS-EU database can be accessed at three levels via www.stressdatabase.eu: 1) meta-data, 2) dynamic data overview, and 3) individual participant data (IPD) (only after analysis plan approval). Anonymized IPD is released via an opt-in principle after analysis plan approval by the STRESS-EU steering committee (Fig. 4). Our web portal (www.stressdatabase.eu) has an interactive interface where researchers can explore meta-data and look at the availability of their variables of interest. Currently five analysis plans have been submitted and approved (see Table 1). The website also lists previously approved analysis plans and describes the procedures to submit an analysis plan. The STRESS-EU database is governed by a consortium agreement (compliant with EU regulations) open to any qualified researcher worldwide who wants to join (www.stressdatabase.eu). Interested researchers can contribute data from their laboratory-based acute stress tests in humans by becoming a formal member of the STRESS-EU consortium. New members have to sign the consortium agreement before they can contribute their data. All data sharing will be done in compliance with EU privacy regulations and original informed consents.

**Table 1:** Approved analysis plans in 2023.

	Researchers	Title	Approval date
1	Hernaus, Quaedflieg	Which sociodemographic, psychophysiological, and experimental variables robustly differentiate cortisol responders from non-responders to acute stress induction?	4/25/2022
2	Broeder, Pasteuning, Sep, Vinkers	Associations between childhood trauma and stress system reactivity	4/25/2022
3	de Nooij, Posthuma, Sep, Quaedflieg, Hernaus, Vinkers, Hermans	Mathematical modelling of the cortisol stress response	9/22/2022
4	Sep, Hermans, de Nooij, Quaedflieg, Hernaus, Broeder, Pasteuning, Vinkers	General pipeline to calculate classical measures of cortisol dynamics in the STRESS-EU database	9/22/2022
5	Sep, Habets, Vinkers	Impact of acute stress paradigms on stress responsivity in healthy and patient populations	9/22/2022

## 6 Future directions and international collaboration

Here we highlighted the STRESS-EU database as a rich European resource of human acute stress studies. This international collaborative initiative offers a wealth of direct (i.e. via the IPD data) and indirect (i.e. via insights from IPD data) scientific advantages to the broader, worldwide, community of stress and resilience researchers.

An example of a direct advantage is that the STRESS-EU database facilitates the possibilities of making use of ‘big data’ for both theory- and data-driven research and the integration of the two (Maass et al., 2018) by bringing together IPD from many individual studies. This opens the possibility for the integration of multiple data types using tools and techniques from computational psychiatry (Huys et al., 2016; Huys et al.; Acosta et al., 2022). Multimodal integration is of specific interest to the community of stress and resilience researchers as the stress response is inherently multimodal, expanding across (neuro)biological, emotional, cognitive and behavioral levels, as are the factors that explain its inter-individual differences and relation to resilience and psychopathology (de Kloet et al., 2005; Joëls et al., 2009; van Oort et al., 2017; Mcewen et al., 2020; Sanacora et al., 2022; Ioannidis et al., 2020).

An example of an initiative sparked by the STRESS-EU database arose from the observation that there is currently no consensus on the best practices in the field of human acute stress studies, which became apparent by the diversity of research methods (e.g. stress-induction paradigms) and datatypes (e.g. stress-related outcomes) in the database. This sparked the ambition within the consortium to embark on a qualitative research project to gather expert opinions, of both (former) participants and researchers, about human acute stress studies and to systematically work towards expert consensus, for example by using the Delphi technique (Barrett et al., 2020). Developing (several) standardized protocols as ‘gold standard’ for human acute stress studies would greatly benefit future studies and improve harmonization within the field.

We aim to expand the database (in Europe and worldwide) and cordially invite all interested researchers worldwide to contribute to and participate in the STRESS-EU database initiative. Further international expansion of the STRESS-EU database is facilitated by the active involvement of the current STRESS-EU consortium members in other international networks, including the Global Stress and Resilience Network (GSRNet), Swiss Stress Network (www.stressnetwork.ch), STRESS-NL consortium (www.stress-nl.nl), International Resilience Alliance (INTRESA) (www.intresa.org), ECNP Traumatic Stress Network (https://www.ecnp.eu/research-innovation/networks-thematic-working-groups/list-ecnp-networks/traumatic-stress), and ECNP Resilience Network (https://www.ecnp.eu/research-innovation/networks-thematic-working-groups/list-ecnp-networks/resilience). Please visit our website (www.stressdatabase.eu) to explore the meta-information of the database, contribute data and/or submit analysis plans.

## Declaration of competing interest

The authors declare that they have no known competing financial interests or personal relationships that could have appeared to influence the work reported in this paper.
