# Supplementary material for: The STRESS-EU database: A European resource of human acute stress studies for the worldwide research community
Source: Neurosci Appl. 2024 Apr 4;3:104063. doi: 10.1016/j.nsa.2024.104063 (PMC12244071; doi:10.1016/j.nsa.2024.104063)
Supplement: Multimedia component 1 [file mmc1.docx]

**Appendix – original papers of studies in the STRESS-EU database**

Bakvis, P., Spinhoven, P., Giltay, E. J., Kuyk, J., Edelbroek, P. M., Zitman, F. G., & Roelofs, K. (2010). Basal hypercortisolism and trauma in patients with psychogenic nonepileptic seizures. Epilepsia, 51(5), 752–759. https://doi.org/10.1111/J.1528-1167.2009.02394.X

Bakvis, P., Spinhoven, P., Zitman, F. G., & Roelofs, K. (2011). Automatic avoidance tendencies in patients with Psychogenic Non Epileptic Seizures. Seizure, 20(8), 628–634. https://doi.org/10.1016/j.seizure.2011.06.006

Bentele, U. U., Meier, M., Benz, A. B. E., Denk, B. F., Dimitroff, S. J., Pruessner, J. C., & Unternaehrer, E. (2021). The impact of maternal care and blood glucose availability on the cortisol stress response in fasted women. Journal of Neural Transmission, 128(9), 1287–1300. https://doi.org/10.1007/S00702-021-02350-Y/TABLES/3

Bouma, E. M. C., Riese, H., Ormel, J., Verhulst, F. C., & Oldehinkel, A. J. (2009). Adolescents’ cortisol responses to awakening and social stress; effects of gender, menstrual phase and oral contraceptives. The TRAILS study. Psychoneuroendocrinology, 34(6), 884–893. https://doi.org/10.1016/J.PSYNEUEN.2009.01.003

Brückl, T. M., Spoormaker, V. I., Sämann, P. G., Brem, A. K., Henco, L., Czamara, D., Elbau, I., Grandi, N. C., Jollans, L., Kühnel, A., Leuchs, L., Pöhlchen, D., Schneider, M., Tontsch, A., Keck, M. E., Schilbach, L., Czisch, M., Lucae, S., Erhardt, A., & Binder, E. B. (2020). The biological classification of mental disorders (BeCOME) study: a protocol for an observational deep-phenotyping study for the identification of biological subtypes. BMC Psychiatry, 20(1). https://doi.org/10.1186/S12888-020-02541-Z

Cornelisse, S., van Stegeren, A. H., & Joëls, M. (2011). Implications of psychosocial stress on memory formation in a typical male versus female student sample. Psychoneuroendocrinology, 36(4), 569–578. https://doi.org/10.1016/J.PSYNEUEN.2010.09.002

Cornelisse, S., Joëls, M., & Smeets, T. (2011). A randomized trial on mineralocorticoid receptor blockade in men: effects on stress responses, selective attention, and memory. Neuropsychopharmacology : Official Publication of the American College of Neuropsychopharmacology, 36(13), 2720–2728. https://doi.org/10.1038/NPP.2011.162

de Brouwer, S. J. M., van Middendorp, H., Stormink, C., Kraaimaat, F. W., Sweep, F. C. G. J., de Jong, E. M. G. J., Schalkwijk, J., Eijsbouts, A., Donders, A. R. T., van de Kerkhof, P. C. M., van Riel, P. L. C. M., & Evers, A. W. M. (2014). The psychophysiological stress response in psoriasis and rheumatoid arthritis. British Journal of Dermatology, 170(4), 824–831. https://doi.org/10.1111/BJD.12697

de Brouwer, S. J. M., Kraaimaat, F. W., Sweep, F. C. G. J., Donders, R. T., Eijsbouts, A., van Koulil, S., van Riel, P. L. C. M., & Evers, A. W. M. (2011). Psychophysiological Responses to Stress after Stress Management Training in Patients with Rheumatoid Arthritis. PLOS ONE, 6(12), e27432. https://doi.org/10.1371/JOURNAL.PONE.0027432

de Rooij, S. R. (2013). Blunted cardiovascular and cortisol reactivity to acute psychological stress: a summary of results from the Dutch Famine Birth Cohort Study. International Journal of Psychophysiology : Official Journal of the International Organization of Psychophysiology, 90(1), 21–27. https://doi.org/10.1016/J.IJPSYCHO.2012.09.011

Giesbrecht, T., Smeets, T., Merckelbach, H., & Jelicic, M. (2007). Depersonalization experiences in undergraduates are related to heightened stress cortisol responses. The Journal of Nervous and Mental Disease, 195(4), 282–287. https://doi.org/10.1097/01.NMD.0000253822.60618.60

Hartman, C. A., Hermanns, V. W., de Jong, P. J., & Ormel, J. (2013). Self- or parent report of (co-occurring) internalizing and externalizing problems, and basal or reactivity measures of HPA-axis functioning: a systematic evaluation of the internalizing-hyperresponsivity versus externalizing-hyporesponsivity HPA-axis hypothesis. Biological Psychology, 94(1), 175–184. https://doi.org/10.1016/J.BIOPSYCHO.2013.05.009

Haushofer, J., Cornelisse, S., Seinstra, M., Fehr, E., Joëls, M., & Kalenscher, T. (2013). No Effects of Psychosocial Stress on Intertemporal Choice. PLOS ONE, 8(11), e78597. https://doi.org/10.1371/JOURNAL.PONE.0078597

Henckens, M. J. A. G., Klumpers, F., Everaerd, D., Kooijman, S. C., van Wingen, G. A., & Fernández, G. (2016). Interindividual differences in stress sensitivity: basal and stress-induced cortisol levels differentially predict neural vigilance processing under stress. Social Cognitive and Affective Neuroscience, 11(4), 663–673. https://doi.org/10.1093/SCAN/NSV149

Hermans, E. J., van Marle, H. J. F., Ossewaarde, L., Henckens, M. J. A. G., Qin, S., van Kesteren, M. T. R., Schoots, V. C., Cousijn, H., Rijpkema, M., Oostenveld, R., & Fernández, G. (2011). Stress-related noradrenergic activity prompts large-scale neural network reconfiguration. Science, 334(6059), 1151–1153. https://doi.org/10.1126/SCIENCE.1209603/SUPPL_FILE/HERMANS.SOM.PDF

Houtepen, L. C., Boks, M. P. M., Kahn, R. S., Joëls, M., & Vinkers, C. H. (2015). Antipsychotic use is associated with a blunted cortisol stress response: a study in euthymic bipolar disorder patients and their unaffected siblings. European Neuropsychopharmacology : The Journal of the European College of Neuropsychopharmacology, 25(1), 77–84. https://doi.org/10.1016/J.EURONEURO.2014.10.005

Jansen, S. W. M., van Heemst, D., van der Grond, J., Westendorp, R., & Oei, N. Y. L. (2016). Physiological responding to stress in middle-aged males enriched for longevity: a social stress study. Stress (Amsterdam, Netherlands), 19(1), 28–36. https://doi.org/10.3109/10253890.2015.1105213

Kaldewaij, R., Koch, S. B. J., Zhang, W., Hashemi, M. M., Klumpers, F., & Roelofs, K. (2019). Frontal Control Over Automatic Emotional Action Tendencies Predicts Acute Stress Responsivity. Biological Psychiatry: Cognitive Neuroscience and Neuroimaging, 4(11), 975–983. https://doi.org/10.1016/J.BPSC.2019.06.011

Klumpers, F., Kroes, M. C., Heitland, I., Everaerd, D., Akkermans, S. E. A., Oosting, R. S., van Wingen, G., Franke, B., Kenemans, J. L., Fernández, G., & Baas, J. M. P. (2015). Dorsomedial prefrontal cortex mediates the impact of serotonin transporter linked polymorphic region genotype on anticipatory threat reactions. Biological Psychiatry, 78(8), 582–589. https://doi.org/10.1016/j.biopsych.2014.07.034

Meier, M., Bentele, U. U., Benz, A. B. E., Denk, B., Dimitroff, S., Pruessner, J. C., & Unternaehrer, E. (2021). Effects of psychological, sensory, and metabolic energy prime manipulation on the acute endocrine stress response in fasted women. Psychoneuroendocrinology, 134, 105452. https://doi.org/10.1016/J.PSYNEUEN.2021.105452

Meier, M., Wirz, L., Dickinson, P., & Pruessner, J. C. (2021). Laughter yoga reduces the cortisol response to acute stress in healthy individuals. Stress (Amsterdam, Netherlands), 24(1), 44–52. https://doi.org/10.1080/10253890.2020.1766018Nelemans, S.A. , Hale, W. W. III, Branje, S., van Lier, P. A. C., Koot, H. M., & Meeus, W. (2017). The role of stress reactivity in the long-term persistence of adolescent social anxiety symptoms. *Biological Psychology,* 125, 91-104. doi: 10.1016/j.biopsycho.2017.03.003

Niermann, H. C. M., Figner, B., Tyborowska, A., van Peer, J. M., Cillessen, A. H. N., & Roelofs, K. (2017). Defensive freezing links Hypothalamic-Pituitary-Adrenal-axis activity and internalizing symptoms in humans. Psychoneuroendocrinology, 82, 83–90. https://doi.org/10.1016/J.PSYNEUEN.2017.05.001

Oei, N. Y. L., Jansen, S. W., Veer, I. M., Slagboom, P. E., van de Grond, J., & van Heemst, D. (2018). Stress evokes stronger medial posterior cingulate deactivations during emotional distraction in slower paced aging. Biological Psychology, 135, 84–92. https://doi.org/10.1016/J.BIOPSYCHO.2018.02.018

Oei, N. Y. L., Everaerd, W. T. A. M., Elzinga, B. M., van Well, S., & Bermond, B. (2006). Psychosocial stress impairs working memory at high loads: An association with cortisol levels and memory retrieval. Stress, 9(3), 133–141. https://doi.org/10.1080/10253890600965773

Oei, N. Y. L., Veer, I. M., Wolf, O. T., Spinhoven, P., Rombouts, S. A. R. B., & Elzinga, B. M. (2012). Stress shifts brain activation towards ventral “affective” areas during emotional distraction. Social Cognitive and Affective Neuroscience, 7(4), 403–412. https://doi.org/10.1093/SCAN/NSR024

Oei, N. Y. L., Both, S., van Heemst, D., & van der Grond, J. (2014). Acute stress-induced cortisol elevations mediate reward system activity during subconscious processing of sexual stimuli. Psychoneuroendocrinology, 39(1), 111–120. https://doi.org/10.1016/J.PSYNEUEN.2013.10.005

Oldehinkel, A. J., Rosmalen, J. G. M., Buitelaar, J. K., Hoek, H. W., Ormel, J., Raven, D., Reijneveld, S. A., Veenstra, R., Verhulst, F. C., Vollebergh, W. A. M., & Hartman, C. A. (2015). Cohort Profile Update: the TRacking Adolescents’ Individual Lives Survey (TRAILS). International Journal of Epidemiology, 44(1), 76–76n. https://doi.org/10.1093/IJE/DYU225

Quaedflieg, C. W. E. M., Meyer, T., & Smeets, T. (2013). The imaging Maastricht Acute Stress Test (iMAST): A neuroimaging compatible psychophysiological stressor. Psychophysiology, 50(8), 758–766. https://doi.org/10.1111/PSYP.12058

Quaedflieg, C. W. E. M., Meyer, T., Smulders, F. T. Y., & Smeets, T. (2015). The functional role of individual-alpha based frontal asymmetry in stress responding. Biological Psychology, 104, 75–81. https://doi.org/10.1016/J.BIOPSYCHO.2014.11.014

Quaedflieg, C. W. E. M., Schwabe, L., Meyer, T., & Smeets, T. (2013). Time dependent effects of stress prior to encoding on event-related potentials and 24 h delayed retrieval. Psychoneuroendocrinology, 38(12), 3057–3069. https://doi.org/10.1016/J.PSYNEUEN.2013.09.002

Roelofs, K., van Peer, J., Berretty, E., Jong, P. de, Spinhoven, P., & Elzinga, B. M. (2009). Hypothalamus-Pituitary-Adrenal Axis Hyperresponsiveness Is Associated with Increased Social Avoidance Behavior in Social Phobia. Biological Psychiatry, 65(4), 336–343. https://doi.org/10.1016/j.biopsych.2008.08.022

Schakel, L., Veldhuijzen, D. S., van Middendorp, H., Prins, C., Drittij, A. M. H. F., Vrieling, F., Visser, L. G., Ottenhoff, T. H. M., Joosten, S. A., & Evers, A. W. M. (2020). An Internet-Based Psychological Intervention With a Serious Game to Improve Vitality, Psychological and Physical Condition, and Immune Function in Healthy Male Adults: Randomized Controlled Trial. Journal of Medical Internet Research, 22(7). <https://doi.org/10.2196/14861>

Sep MSC, Joëls M, Geuze E. Individual differences in the encoding of contextual details following acute stress: An explorative study. Eur J Neurosci. 2022 May;55(9-10):2714-2738. doi: 10.1111/ejn.15067.

Smeets, T., Cornelisse, S., Quaedflieg, C. W. E. M., Meyer, T., Jelicic, M., & Merckelbach, H. (2012). Introducing the Maastricht Acute Stress Test (MAST): a quick and non-invasive approach to elicit robust autonomic and glucocorticoid stress responses. Psychoneuroendocrinology, 37(12), 1998–2008. https://doi.org/10.1016/J.PSYNEUEN.2012.04.012

Smeets, T., Dziobek, I., & Wolf, O. T. (2009). Social cognition under stress: Differential effects of stress-induced cortisol elevations in healthy young men and women. Hormones and Behavior, 55(4), 507–513. https://doi.org/10.1016/j.yhbeh.2009.01.011

Smeets, T., Jelicic, M., Merckelbach, H., Peters, M., Fett, A., Taverniers, J., Henquet, C., & Dautzenberg, J. (2006). Enhanced memory performance on an internal-internal source monitoring test following acute psychosocial stress. Behavioral Neuroscience, 120(6), 1204–1210. https://doi.org/10.1037/0735-7044.120.6.1204

Smeets, T., Otgaar, H., Raymaekers, L., Peters, M. J. V., & Merckelbach, H. (2012). Survival processing in times of stress. Psychonomic Bulletin and Review, 19(1), 113–118. https://doi.org/10.3758/S13423-011-0180-Z/TABLES/1

Smeets, T., Jelicic, M., & Merckelbach, H. (2006). Stress-induced cortisol responses, sex differences, and false recollections in a DRM paradigm. Biological Psychology, 72(2), 164–172. https://doi.org/10.1016/J.BIOPSYCHO.2005.09.004

Smeets, T., Giesbrecht, T., Jelicic, M., & Merckelbach, H. (2007). Context-dependent enhancement of declarative memory performance following acute psychosocial stress. Biological Psychology, 76(1–2), 116–123. https://doi.org/10.1016/J.BIOPSYCHO.2007.07.001

Smeets, T. (2010). Autonomic and hypothalamic–pituitary–adrenal stress resilience: Impact of cardiac vagal tone. Biological Psychology, 84(2), 290–295. https://doi.org/10.1016/J.BIOPSYCHO.2010.02.015

Smeets, T., Jelicic, M., & Merckelbach, H. (2006). The effect of acute stress on memory depends on word valence. International Journal of Psychophysiology, 62(1), 30–37. https://doi.org/10.1016/J.IJPSYCHO.2005.11.007

Smeets, T., Wolf, O. T., Giesbrecht, T., Sijstermans, K., Telgen, S., & Joëls, M. (2009). Stress selectively and lastingly promotes learning of context-related high arousing information. Psychoneuroendocrinology, 34(8), 1152–1161. https://doi.org/10.1016/J.PSYNEUEN.2009.03.001

Smeets, T. (2011). Acute stress impairs memory retrieval independent of time of day. Psychoneuroendocrinology, 36(4), 495–501. https://doi.org/10.1016/J.PSYNEUEN.2010.08.001

Tekampe, J., van Middendorp, H., Biermasz, N. R., Sweep, F. C. G. J., Meijer, O. C., Pelsma, I. C. M., Pereira, A. M., Hermus, A. R. M. M., & Evers, A. W. M. (2021). Conditioning cortisol in healthy young women – A randomized controlled trial. Psychoneuroendocrinology, 124, 105081. https://doi.org/10.1016/J.PSYNEUEN.2020.105081

Tollenaar, M. S., Elzinga, B. M., Spinhoven, P., & Everaerd, W. A. M. (2008). The effects of cortisol increase on long-term memory retrieval during and after acute psychosocial stress. Acta Psychologica, 127(3), 542–552. https://doi.org/10.1016/J.ACTPSY.2007.10.007

Tollenaar, M. S., & Overgaauw, S. (2017). Empathy and mentalizing abilities in relation to psychosocial stress in healthy adult men and women. Heliyon, e04488. https://doi.org/10.1016/j.heliyon.2020.e04488

Tollenaar, M. S., Elzinga, B. M., Spinhoven, P., & Everaerd, W. (2009). Autobiographical memory after acute stress in healthy young men. Memory (Hove, England), 17(3), 301–310. https://doi.org/10.1080/09658210802665845

van Campen, J. S., Jansen, F. E., Pet, M. A., Otte, W. M., Hillegers, M. H. J., Joels, M., & Braun, K. P. J. (2015). Relation between stress-precipitated seizures and the stress response in childhood epilepsy. Brain : A Journal of Neurology, 138(Pt 8), 2234–2248. https://doi.org/10.1093/BRAIN/AWV157

Veer, I. M., Oei, N. Y. L., Spinhoven, P., van Buchem, M. A., Elzinga, B. M., & Rombouts, S. A. R. B. (2011). Beyond acute social stress: Increased functional connectivity between amygdala and cortical midline structures. NeuroImage, 57(4), 1534–1541. https://doi.org/10.1016/J.NEUROIMAGE.2011.05.074

Vinkers, C. H., Zorn, J. v., Cornelisse, S., Koot, S., Houtepen, L. C., Olivier, B., Verster, J. C., Kahn, R. S., Boks, M. P. M., Kalenscher, T., & Joëls, M. (2013). Time-dependent changes in altruistic punishment following stress. Psychoneuroendocrinology, 38(9), 1467–1475. https://doi.org/10.1016/J.PSYNEUEN.2012.12.012

Voulgaropoulou, S. D., Fauzani, F., Pfirrmann, J., Vingerhoets, C., van Amelsvoort, T., & Hernaus, D. (2022). Asymmetric effects of acute stress on cost and benefit learning. Psychoneuroendocrinology, 138, 105646. https://doi.org/10.1016/J.PSYNEUEN.2021.105646

Wirz, L., Wacker, J., Felten, A., Reuter, M., & Schwabe, L. (2017). A Deletion Variant of the α2b-Adrenoceptor Modulates the Stress-Induced Shift from “Cognitive” to “Habit” Memory. Journal of Neuroscience, 37(8), 2149–2160. https://doi.org/10.1523/JNEUROSCI.3507-16.2017

Zhang, W., Hashemi, M. M., Kaldewaij, R., Koch, S. B. J., Beckmann, C., Klumpers, F., & Roelofs, K. (2019). Acute stress alters the ‘default’ brain processing. NeuroImage, 189, 870–877. https://doi.org/10.1016/J.NEUROIMAGE.2019.01.063
